# Clinical characteristics and prognosis of patients with glioblastoma: A review of survival analysis of 1674 patients based on SEER database

**DOI:** 10.1097/MD.0000000000032042

**Published:** 2022-11-25

**Authors:** Ligang Chen, Jing Ma, Zheng Zou, Hongzhe Liu, Chenxin Liu, Shun Gong, Xu Gao, Guobiao Liang

**Affiliations:** a Department of Neurosurgery, General Hospital of Northern Theater Command, Shenyang, China; b Department of Pathology, Xijing Hospital, Air Force Medical University, Xi’an, Shaanxi, China; c Department of Pediatric Orthopedics, Honghui Hospital, Xi’an Jiaotong University, Xi’an, Shaanxi, China.

**Keywords:** GBM, prognosis, risk factor, SEER database, survival

## Abstract

To fully understand the clinical features and prognosis of Glioblastoma (GBM), we extracted the data from the Surveillance, Epidemiology, and End Results (SEER) database and performed a series of analyses.

**Methods:**

We retrospectively analyzed the data of 1674 patients with GBM obtained from the SEER database from 1983 to 2015. Kaplan–Meier analysis was performed to calculate the survival rate, and the log-rank test was used to analyze the survival outcomes.

**Results:**

Older patients with GBM had a worse survival period (*P* < .05). Laterality had no effect on the prognosis (*P* > .05). Patients with high-grade gliomas may have a shorter lifespan (*P* < .05). In terms of overall survival (OS) and disease specificity, all 3 classical treatments failed to improve the life expectancy (*P* > .05). In adult patients with GBM, we found that age, tumor grade, surgery, radiotherapy, and chemotherapy were independent risk factors for all-cause mortality. In the univariate disease-specific analysis, age, tumor grade, surgery, radiotherapy, and chemotherapy were independent risk factors. However, in multivariate disease-specific analysis, the results showed that only tumor grade and surgery were independent risk factors for GBM.

**Conclusions:**

Older patients diagnosed with GBM have worse survival, and patients with glioma of higher grades have a shorter lifespan. Age, grade, surgery, radiation therapy, and chemotherapy were independent prognostic factors for patients with GBM.

## 1. Introduction

Glioblastoma (GBM) is the most malignant brain tumor, with high morbidity and mortality.^[1]^ In the United States (US) population, GBM is associated with advanced age and male sex^[2]^ and is more frequently observed in Caucasians than in other ethnicities.^[3]^ GBM is disproportionately associated with high morbidity and mortality, with a 5-year overall survival (OS) of only 7.2%, varying by age and sex.^[4,5]^ GBM patients exhibit rapid expansion or destruction of brain structures, accompanied by intertwined nerve symptoms, including focal neurological signs, mental status alterations, and signs of increased intracranial pressure.^[6]^ Previous studies have shown that risk factors for GBM account for only a small proportion of cases.^[7]^ Exposure to non-ionizing radiation, mostly due to cell phone usage, is considered a potential risk factor for brain tumors; however, no consistent evidence has been found yet.^[8]^

GBM is diagnosed on the basis of clinical and radiological assessments. Contrast-enhanced magnetic resonance imaging (MRI) is an important tool for the diagnosis of GBM. However, other types of intra-axial neoplasms, such as metastasized or some lower grade gliomas, along with other non-neoplastic neurological conditions, could interfere with GBM diagnosis when using MRI.^[9–11]^ Currently, the standard of care for GBM consists of extended surgical resection, radiotherapy, and chemotherapy.^[12–14]^ Surgical resection, including gross total resection (GTR), is positively correlated with survival time in patients with GBM.^[15]^ Radiation therapy (RT) uses X-ray photons, gamma photons, protons, and stereotactic radiosurgery (SRS).^[16]^ However, none of these therapies benefit patients in the clinic. Classical chemotherapy for GBM involves cytotoxic chemotherapy using the alkylating agent temozolomide (TMZ).^[17,18]^ TMZ has been used for postsurgical treatment and combined radiochemotherapy. Several new compounds, including carmustine (BCNU), have been explored for the treatment of GBM; however, they have only been used to ensure therapeutic efficacy.^[19]^

GBM is the most lethal brain tumor, with limited treatment options. There is no doubt that epidemiological and etiological data are vital for treating this type of disease. Herein, we retrospectively analyzed the data of patients with GBM from the Surveillance, Epidemiology, and End Results (SEER) database and performed a series of analyses to fully understand the clinical features, prognosis, and their association with risk factors for GBM.

## 2. Methods

The SEER database is a population-based cancer registry supported by the National Cancer Institute of US, covering approximately 28% of the US population. The database holds annually uploaded data on patient demographics, tumor pathology, anatomic sites of the tumor, stage at diagnosis, first course of treatment modalities, and follow-up vital status. In this study, the clinical data of patients with GBM registered between 1983 and 2015 were downloaded from the database. Due to public availability and anonymized patient information, this study was exempted from obtaining approval from the institutional review board.

**Figure 1.**
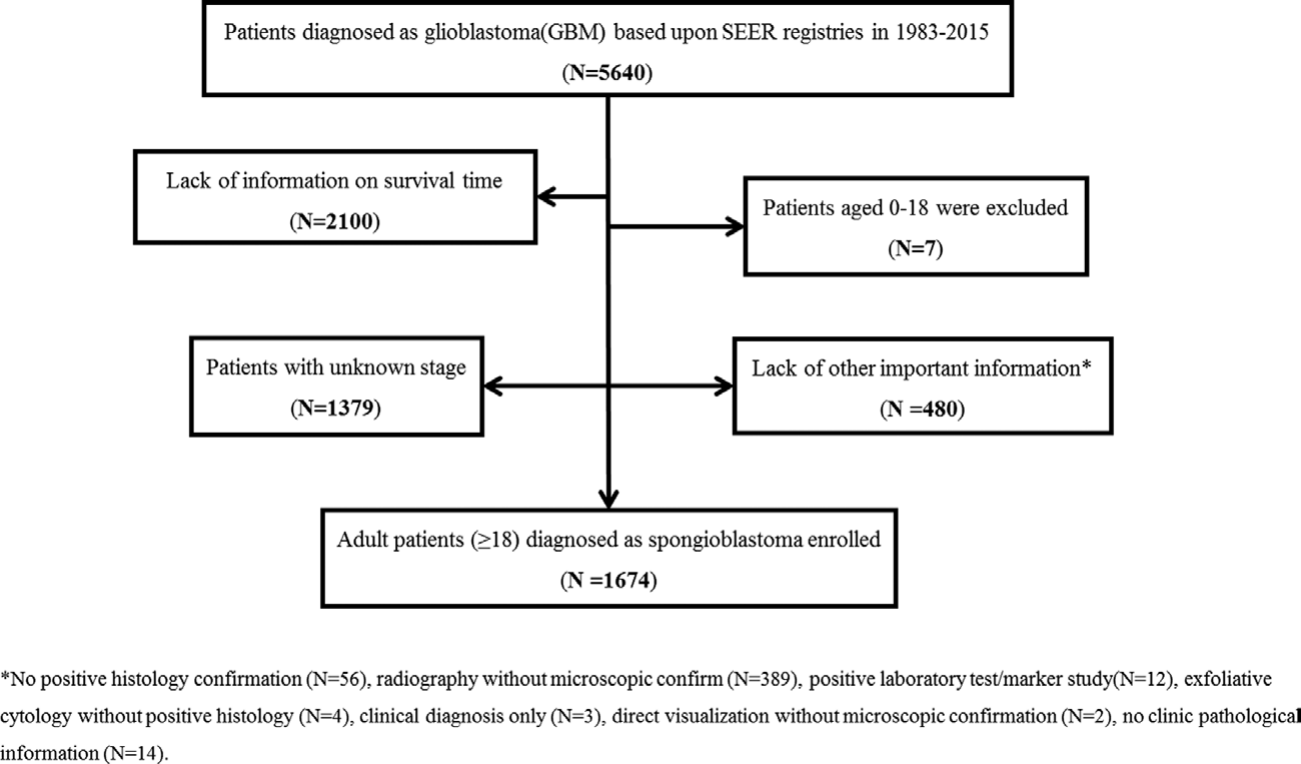
The flowchart of data identification process.

The patient selection process is illustrated in Figure 1. A total of forty GBM patients were identified, among whom 1674 cases (29.68%) with complete survival information were selected for further analysis. The inclusion criteria were as follows: primary tumor localized in the brain (ICD-O-3: C71.0-9), and histological type restricted to GBM. The exclusion criteria were as follows: patients with no survival information, patients aged < 18 years (due to potentially different natural history of the disease), patients with unclear stage information, and patients with no important clinicopathological information.

All-cause mortality was calculated as the period between pathological diagnosis and the last follow-up or death due to any cause. Disease (cancer) specific survival (DSS) was defined as the duration between diagnosis and the last follow-up or death due to GBM. Causes of death are listed in Supplementary Table 1, http://links.lww.com/MD/I12.

SPSS (version 26.0) was used to perform statistical analyses. The influence of the variables on survival outcomes was assessed using the log-rank test and survival curves. The Cox proportional hazards regression model was used for the multivariate analysis of DSS and all-cause mortality, and variables with statistical significance in the univariate analysis were selected. All tests were considered statistically significant for 2-tailed tests, with *P* < .05.

## 3. Results

Herein, we analyzed the data of 1674 patients with GBM, which included 1038 men and 636 women (1.632:1). The demographic characteristics of patients are presented in Table 1. The most frequently affected age group was 60 to 80 years (N = 1054, 63.0%), followed by the 40 to 60 years age group (N = 321, 19.2%). Since the source of the data was hospitals within the US, Caucasians dominated the cohort (N = 1525, 91.1%). Moreover, high-grade (III–IV) GBM was the most frequently observed (N = 1598, 95.5%) in the cohort. As shown in Table 1, most patients had unilateral GBM (N = 746, 44.6%), while bilateral tumors were rarely observed in the cohort (N = 18, 1.1%). Surgery was performed in 79.5% (1331/1674) of patients, and 72.7% (1217/1674) of patients received radiation therapy. Less than half (N = 801, 47.8%) of the patients received chemotherapy according to the database.

**Table 1:** Cohort demographics.

Characteristic	Case (n)	Ratio (%)
**Gender**		
Male	1038	62
Female	636	38
**Age**		
18–40	49	2.9
40–60	321	19.2
60–80	1054	63.0
80-	250	14.9
**Race**		
White	1525	91.1
Black	92	5.5
Asian or pacific islander	57	3.4
**Grade**		
Grade 1–2	76	4.5
Grade 3–4	1598	95.5
**Laterality**		
Left-origin of primary	454	27.1
Right-origin of primary	456	27.2
Bilateral	18	1.1
Not a paired site	746	44.6
**Surgery**		
No	343	20.5
Yes	1331	79.5
**Radiation**		
No	457	27.3
Yes	1217	72.7
**Chemotherapy**		
No	873	52.2
Yes	801	47.8
**Overall survival**		
Censored	119	7.1
Dead	1555	92.9
**Disease-specific survival**		
Censored	120	7.2
Dead	1553	92.8
**Year of diagnosis**		
1975–1985	454	27.1
1985–1995	456	27.2
1995–2005	18	1.1
2005–2016	746	44.6

**Figure 2.**
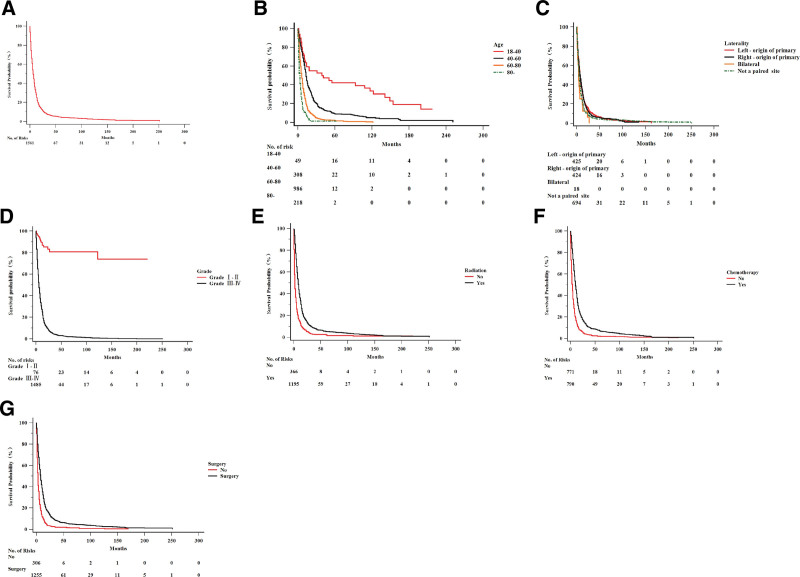
The influence of clinical and therapeutic variables on OS survival of GBM. (A) OS and numbers of risks of the total 1674 cases; (B) OS survival curve on age group; (C) OS survival curve on laterality; (D) OS survival curve on grade; (E) OS survival curve on radiation therapy; (F) OS survival curve on chemotherapy; (G) OS survival curve on surgery. GBM = glioblastoma, OS = overall survival.

The database provided censored rates of 7.1% (N = 119) for OS and 7.2% (N = 120) for DSS. By comparing the Kaplan–Meier survival curves, we assessed the influence of clinical and therapeutic variables on the survival of patients with GBM. The OS and number of risk factors for the analyzed 1674 cases are shown in Figure 2A. In the age group analysis, the OS worsened as age increased (*P* < .001) (Fig. 2B). In laterality analyses, no significant differences were observed between subgroups, yet rare bilateral cases showed the poorest survival (*P* < .05) (Fig. 2C). Significant differences in OS were observed between the higher-grade (III–IV) and lower-grade (I–II) groups (*P* < .0001) (Fig. 2D). Regarding therapeutic variables, all 3 commonly used therapies significantly improved patient survival (*P* < .05) (Fig. 2E–G).

Univariate analysis of all-cause mortality showed that age, laterality, grade, surgery, radiation therapy, and chemotherapy were the independent prognostic factors for GBM (Table 2). Regarding clinical characteristics, older patients had a significant all-cause survival disadvantage (HR = 2.185, 95% CI [1.528–3.124]; HR = 4.333, 95% CI [3.052–6.153]; HR = 7.714, 95% CI [5.326–11.172]) when compared to the 18 to 40 years age group. Patients with a higher stage (Stage III–IV) glioma at diagnosis were at a higher risk of death than those with lower stage (Stage I–II) glioma (HR = 15.151, 95% CI [8.928–25.713]). All 3 therapeutic variables (surgical treatment, radiotherapy, and chemotherapy) were efficient in improving patient survival (*P* < .0001, HR = 0.494, 95% CI [0.437–0.559]; HR = 0.920, 95% CI [0.784–1.079]; and HR = 0.472, 95% CI [0.422–0.528], respectively). We also analyzed the multivariate Cox proportional hazards of all-cause mortality in patients with GBM (Table 3). Consistent with the univariate analysis, the results showed that age, grade, surgery, radiation, and chemotherapy, but not literality, were independent prognostic factors for GBM. Compared to the 18 to 40 years age group, older patients had a significant all-cause survival disadvantage (60–80 years age group HR = 3.188, 95% CI [2.241–4.534]; >80 years age group, HR = 4.299, 95% CI [5.326–11.172]). Patients with stage III to IV glioma were at a higher risk of death than those with stage I to II glioma (HR = 13.195, 95% CI [7.738–22.500]). All 3 therapeutic variables were associated with improved survival of patients with GBM (*P* < .0001) (HR = 0.583, 95% CI [0.514–0.661], HR = 0.920, 95% CI [0.784–1.079], HR = 0.596, 95% CI [0.526–0.675] for surgical treatment, radiotherapy, and chemotherapy, respectively).

**Table 2:** Univariate Cox proportional hazards analysis of all-cause mortality by prognostic factors in adult patients with glioblastoma.

Characteristic	HR (95%CI)	*P*
**Gender**		
Male	Reference	.647
Female	0.976 (0.881–1.082)	
**Age**		
18–40	Reference	**.000**
40–60	2.185 (1.528–3.124)	.021
60–80	4.333 (3.052–6.153)	.010
80-	7.714 (5.326–11.172)	.000
**Race**		
White	Reference	.113
Black	1.010 (0.808–1.262)	
Asian or pacific islander	0.737 (0.552–0.982)	
**Grade**		
Grade 1–2	Reference	**.000**
Grade 3–4	15.151 (8.928–25.713)	
**Laterality**		
Left - origin of primary	Reference	.001
Right - origin of primary	1.018 (0.887–1.169)	.080
Bilateral	1.533 (0.943–2.491)	.059
Not a paired site	1.227 (1.086–1.387)	.125
**Surgery**		
No	Reference	**.000**
Yes	0.494 (0.437–0.559)	
**Radiation**		
No	Reference	**.000**
Yes	0.472 (0.422–0.528)	
**Chemotherapy recode**		
No	Reference	**.000**
Yes	0.501 (0.453–0.555)	
**Year at diagnosis**		
1975–1985	Reference	.499
1985–1995	0.824 (0.552–1.230)	.073
1995–2005	0.948 (0.652–1.379)	.354
2005–2016	0.931 (0.643–1.348)	.200

HR = hazard ratio.

**Table 3:** Multivariate Cox proportional hazards analysis of all-cause mortality by prognostic factors in adult patients with glioblastoma.

Characteristic	HR (95%CI)	*P*
**Age**		
20–40	Reference	**.000**
40–60	1.618 (1.129–2.317)	.009
60–80	3.188 (2.241–4.534)	.000
80-	4.299 (2.958–6.249)	.000
**Laterality**		
Bilateral, single primary	Reference	.081
Left-origin of primary	1.201 (1.044–1.380)	
Right-origin of primary	1.170 (0.718–1.905)	
Other	1.084 (0.951–1.235)	
**Grade**		
Grade I–II	Reference	**.000**
Grade III–IV	13.195 (7.738–22.500)	
**Surgery**		
No	Reference	**.000**
Yes	0.583 (0.514–0.661)	
**Radiation**		
No	Reference	**.000**
Yes	0.920 (0.784–1.079)	
**Chemotherapy**		
No	Reference	**.000**
Yes	0.596 (0.526–0.675)	

HR = hazard ratio.

**Figure 3.**
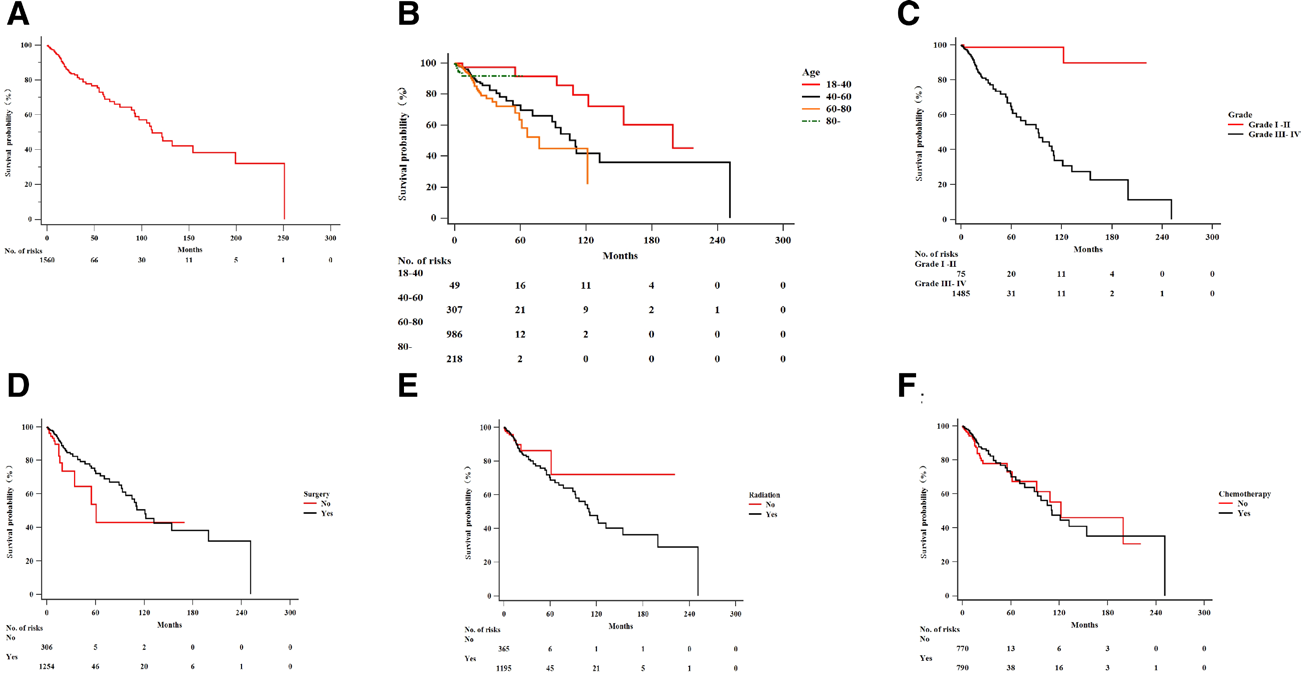
The influence of clinical and therapeutic variables on DSS survival of GBM. (A) DSS and numbers of risks of the total 1674 cases; (B) OS survival curve on age group; (C) OS survival curve on grade; (D) OS survival curve on surgery; (E) OS survival curve on radiation therapy; (F) OS survival curve on chemotherapy. DSS = disease specific survival, GBM = glioblastoma, OS = overall survival.

Figure 3A shows the results of DSS mortality analysis and the number of risk factors in the cohort. Consistent with the OS analysis, we observed that older patients had a poorer prognosis than younger patients (Fig. 3B). Due to limited cases and censored data, patients aged > 80 years showed no difference from other age groups (*P* = .09). Patients with higher-grade glioma had shorter survival periods than those with lower grades in the DSS analysis (*P* < .0001) (Fig. 3C). Contrary to the results of OS analysis, the DSS survival analysis of the therapeutic variables showed that there are no significant differences between patients who received the treatment and those who did not (*P* = .08, *P* = .06, and *P* = .10 for surgery, radiotherapy, and chemotherapy, respectively) (Fig. 3D–F).

Univariate Cox proportional hazards analysis of DSS in GBM patients showed that age, grade, surgery, radiation therapy, and chemotherapy were independent prognostic factors (Table 4). Older patients had a significant survival disadvantage (*P* = .007) (HR = 2.675, 95% CI [1.165–6.143];HR = 3.632, 95% CI [1.544–8.545]; HR = 5.708, 95% CI [2.043–15.945]). Patients with Stage III to IV GBM had poorer survival compared to those with Stage I to II glioma (HR = 15.523, 95% CI [3.788–63.616]). Surgical treatment, radiotherapy, and chemotherapy were associated with long-term survival of GBM patients (*P* < .05) (HR = 0.512, 95% CI [0.317–0.827]; HR = 0.472, 95% CI [0.422–0.528]; HR = 0.994, 95% CI [0.585–1.691, respectively]). The results of the multivariate Cox proportional hazards analysis of DSS are shown in Table 5. Contrary to the results of the univariate analysis, multivariate analysis revealed that only grade and surgery were independent prognostic factors for GBM patients (HR = 13.812, 95% CI [3.333–57.242]; HR = 0.566, 95% CI [0.349–0.917]), but not age, radiotherapy, and chemotherapy.

**Table 4:** Univariate Cox proportional hazards analysis of disease specific mortality by prognostic factors in adult patients with glioblastoma.

Characteristic	HR (95%CI)	*P*
**Gender**		
Male	Reference	.242
Female	0.797 (0.544–1.166)	
**Age**		
18–40	Reference	**.007**
40–60	2.675 (1.165–6.143)	
60–80	3.632 (1.544–8.545)	
80-	5.708 (2.043–15.945)	
**Race**		
White	Reference	.407
Black	0.578 (0.213–1.571)	
Asian or pacific islander	0.651 (0.239–1.769)	
**Grade**		
Grade I–II	Reference	**.000**
Grade III–IV	15.523 (3.788–63.616)	
**Laterality**		
Left-origin of primary	Reference	.361
Right-origin of primary	1.122 (0.664–1.896)	
Bilateral	1.637 (0.841–2.476)	
Not a paired site	1.471 (0.931–2.323)	
**Surgery**		
No	Reference	**.006**
Yes	0.512 (0.317–0.827)	
**Radiation**		
No	Reference	**.007**
Yes	0.472 (0.422–0.528)	
**Chemotherapy**		
No	Reference	**.035**
Yes	0.994 (0.585–1.691)	
**Year of diagnosis**		
1975–1985	Reference	.913
1985–1995	2.163 (0.279–16.764)	
1995–2005	2.374 (0.323–17.441)	
2005–2016	2.469 (0.341–17.899)	

HR = hazard ratio.

**Table 5:** Multivariate cox proportional hazards analysis disease specific survival by prognostic factors in adult patients with glioblastoma.

Characteristic	HR (95%CI)	*P*
**Age**		
20–40	Reference	.065
40–60	1.953 (0.835–4.569)	.123
60–80	2.620 (1.090–6.299)	.071
80-	3.889 (1.350–11.208)	.112
**Grade**		
Grade I–II	Reference	**.000**
Grade III–IV	13.812 (3.333–57.242)	
**Surgery**		
No	Reference	**.021**
Yes	0.566 (0.349–0.917)	
**Radiation**		
No	Reference	.494
Yes	1.221 (0.689–2.165)	
**Chemotherapy**		
No	Reference	.533
Yes	0.665 (0.442–1.000)	

HR = hazard ratio.

## 4. Discussion

GBM is the most malignant form of primary brain tumor with an extremely poor prognosis. Adult GBM patients with classical treatment have a 5-year survival rate of less than 10%. Our results suggest that patients diagnosed with GBM at an older age have a shorter survival period. Moreover, patients with high-grade gliomas (grades III–IV) have a shorter lifespan. Age, tumor grade, surgery, radiation therapy, and chemotherapy were independent prognostic factors for GBM in the OS analysis. Grade and surgery were independent prognostic factors for GBM in the DSS analysis.

Previous studies have shown that anthropometric parameters, such as sex, age, weight, height, and anatomical location of the tumor, are risk factors for cancer.^[20–22]^ Our results showed an increased incidence of GBM in males and Caucasians, which is consistent with the results of previous studies.^[23,24]^ This indicates the potential role of sex hormones in GBM. However, further studies will help in understanding the pathogenesis of this disease. To our knowledge, no previous study has shown that sex is a prognostic factor for patients with GBM.

Studies have revealed that GBM primarily affects elderly patients.^[25–28]^ In our study, an increased incidence of GBM was observed in elderly patients aged 60-80 years age group. We also found that older age was associated with worse prognosis for both OS and DSS in univariate and multivariate analyses. In our study, although only 250 patients were > 80 years of age, we observed that age was not a risk factor for GBM prognosis in the DSS multivariate analysis. However, our results suggest that male patients with GBM have shorter lifespans.

Before 2016, the diagnosis and grading of GBM were based on histopathological analyses. Consistent with existing data,^[25–28]^ our results indicate that tumor grade is an important independent prognostic factor for GBM patients in both OS and DSS by univariate and multivariate analyses. In recent years, novel molecular markers of GBM have been explored, and an advanced grading system based on these molecular markers would help further our understanding of the disease.

Regarding the location of the tumor, our findings suggest that most patients with GBM had unilateral tumors in either the left or right brain hemisphere. In the OS and DSS analyses, no significant differences were observed between subgroups, yet rare bilateral cases showed the poorest survival. Our findings indicated that tumor location might not affect the prognosis of patients with GBM. Excluding all 18 bilateral metastatic cases, it is conceivable that GBM patients with tumor sites within the brain have the shortest survival period.

As the most aggressive type of brain tumor, GBM remains untreatable, and surgery, radiotherapy, chemotherapy, or their combination failed to yield satisfactory results and improve the survival time.^[23,29,30]^ Our data on GBM patients from 1983 to 2015 suggests that surgery, radiotherapy, and chemotherapy are inefficient in improving the prognosis of patients with GBM. In fact, with incomplete resection, the recurrence rate of GBM has remained high over the past decades.^[31]^ Based on studies of molecular markers, immunotherapy, GBM stem cells, and advanced MRI, several novel treatment strategies for GBM have been developed. These strategies include targeted molecular (precision) therapies^[32]^ targeting DNA damage response (DDR) pathways,^[33]^ tumor metabolism,^[34]^ immunotherapies,^[35]^ and viral therapies.^[36]^ However, only a few of these therapies have yielded satisfactory results. Further studies on the underlying mechanisms are required to better understand GBM pathogenesis.

Our study had several limitations. First, we did not include family history, blood test results, immunohistochemistry results, MRI findings, international prognostic index (IPI) score, data on recurrence, and presence of genetic mutations in our analysis because this information was lacking in the SEER records. Second, the treatment variables that influenced prognosis could not be fully evaluated because details of the surgical procedures, chemotherapy regimens and doses, and radiation dose/technology were not included in the SEER database. Third, although information from 1983 to 2015 was retrieved from the SEER database, diagnostic criteria are changing with the development of novel molecular markers and therapeutic methods.

## 5. Conclusion

Patients with GBM diagnosed at an older age tended to have a worse survival period. Higher grades (grade III–IV) of GBM may reduce the lifespan of patients. In addition to surgery, classical radiotherapy and chemotherapy failed to improve the survival time of GBM patients. Age, grade, surgery, radiation therapy, and chemotherapy were independent prognostic factors of GBM patients with GBM in the OS analysis. Grade and surgery were independent prognostic factors for patients with GBM in the DSS analysis.

## Acknowledgments

Great appreciation should be accorded to all the researchers and staff of the Surveillance, Epidemiology, and End Results (SEER) Program for their hard work in collecting patient information and maintaining the database. This study was funded by the National Natural Science Foundation of China (no. 81971133).

## Author contributions

Shun Gong, Xu Gao, and Guobiao Liang contributed to the conception of this study and provided constructive discussions. Hongzhe Liu and Chenxin Liu contributed to data collection; Ligang Chen, Jing Ma, and Zheng Zou contributed to data analysis and manuscript preparation.

**Conceptualization:** Ligang Chen, Chenxin Liu, Shun Gong.

**Data curation:** Ligang Chen, Jing Ma, Chenxin Liu.

**Formal analysis:** Zheng Zou, Hongzhe Liu.

**Funding acquisition:** Xu Gao.

**Investigation:** Jing Ma, Hongzhe Liu.

**Methodology:** Shun Gong.

**Project administration:** Xu Gao.

**Resources:** Guobiao Liang.

**Software:** Jing Ma, Zheng Zou.

**Supervision:** Chenxin Liu, Shun Gong, Xu Gao, Guobiao Liang.

**Visualization:** Zheng Zou, Guobiao Liang.

**Writing – original draft:** Ligang Chen, Shun Gong.

**Writing – review & editing:** Ligang Chen, Shun Gong, Guobiao Liang.

## Supplementary Material
