# Supplementary material for: Clinical characteristics and prognosis of patients with glioblastoma: A review of survival analysis of 1674 patients based on SEER database
Source: Medicine (Baltimore). 2022 Nov 25;101(47):e32042. doi: 10.1097/MD.0000000000032042 (PMC9704894; doi:10.1097/MD.0000000000032042)
Supplement: Supplementary file 1 [file medi-101-e32042-s001.pdf]

Supplemental Table 1: Distribution of cause of death.

Reasons of death	No.	Percentage
Accidents and Adverse Effects	4	0.2%
Aleukemic, Subleukemic and NOS	2	0.1%
Alive	119	7.1%
Atherosclerosis	1	0.1%
Brain and Other Nervous System	1251	74.7%
Breast	17	1.0%
Cerebrovascular Diseases	9	0.5%
Chronic Lymphocytic Leukemia	2	0.1%
Chronic Obstructive Pulmonary Disease and Allied Cond	5	0.3%
Colon excluding Rectum	11	0.7%
Diabetes Mellitus	4	0.2%
Diseases of Heart	19	1.1%
Esophagus	1	0.1%
Hodgkin Lymphoma	1	0.1%
In situ, benign or unknown behavior neoplasm	78	4.7%
Kidney and Renal Pelvis	4	0.2%
Liver	1	0.1%
Lung and Bronchus	21	1.3%
Melanoma of the Skin	4	0.2%
Miscellaneous Malignant Cancer	33	2.0%
Non-Hodgkin Lymphoma	2	0.1%
Oropharynx	1	0.1%
Other Cause of Death	22	1.3%
Other Digestive Organs	1	0.1%
Other Diseases of Arteries, Arterioles, Capillaries	1	0.1%
Other Infectious and Parasitic Diseases including HIV	7	0.4%
Other Myeloid/Monocytic Leukemia	1	0.1%
Ovary	4	0.2%
Pancreas	1	0.1%
Pneumonia and Influenza	9	0.5%
Prostate	14	0.8%
Rectum and Rectosigmoid Junction	2	0.1%
Septicemia	2	0.1%
State DC not available or state DC available but no COD	7	0.4%
Stomach	2	0.1%
Symptoms, Signs and Ill-Defined Conditions	1	0.1%
Thyroid	4	0.2%
Urinary Bladder	6	0.4%
Total	1674	100.0
